# Career perspective: Jim Milledge

**DOI:** 10.1186/2046-7648-1-9

**Published:** 2012-11-07

**Authors:** James Sibree Milledge

**Affiliations:** 1 CASE Medicine, Portex Unit, UCL Institute of Child Health, 30 Guilford Street, London, WC1N 1EH, UK

**Keywords:** Autobiography, Altitude physiology medicine, High altitude

## Abstract

This paper is an overview of my career as a hospital physician with special interest in respiratory diseases. Alongside this career, I have been fortunate to be able to pursue my professional hobby of high altitude medicine and physiology, partly in the laboratory but mainly in the field in the great ranges of the world.

## My career, in brief

I was born in 1930 in China, where my father was a medical missionary. I came to the UK in 1936 and was brought up in North Wales where I started hillwalking in Snowdonia. In 1948, I entered Birmingham Medical School where I got the rock-climbing bug.

After graduating and house jobs, I had to do national service and joined the Royal Air Force (RAF) as a medical officer. I was posted in Hong Kong. The commanding officer was an Alpine Club member, and we formed a mountain rescue team. Besides climbing any rock we could find, we had an expedition to Borneo, to Kinabalu—my first expedition—in 1957. The following year, my wife and I had a 2-week trek in Nepal. This was entirely self-organised with just two porters, with no common language. We fell in love with the country.

After demobilisation from the RAF, I decided to specialise in general medicine with an interest in respiratory disease. Working in Southampton with William McLeod, we performed arterial blood sampling in the investigation of lung disease—cutting edge science at the time!

**Figure 1:**
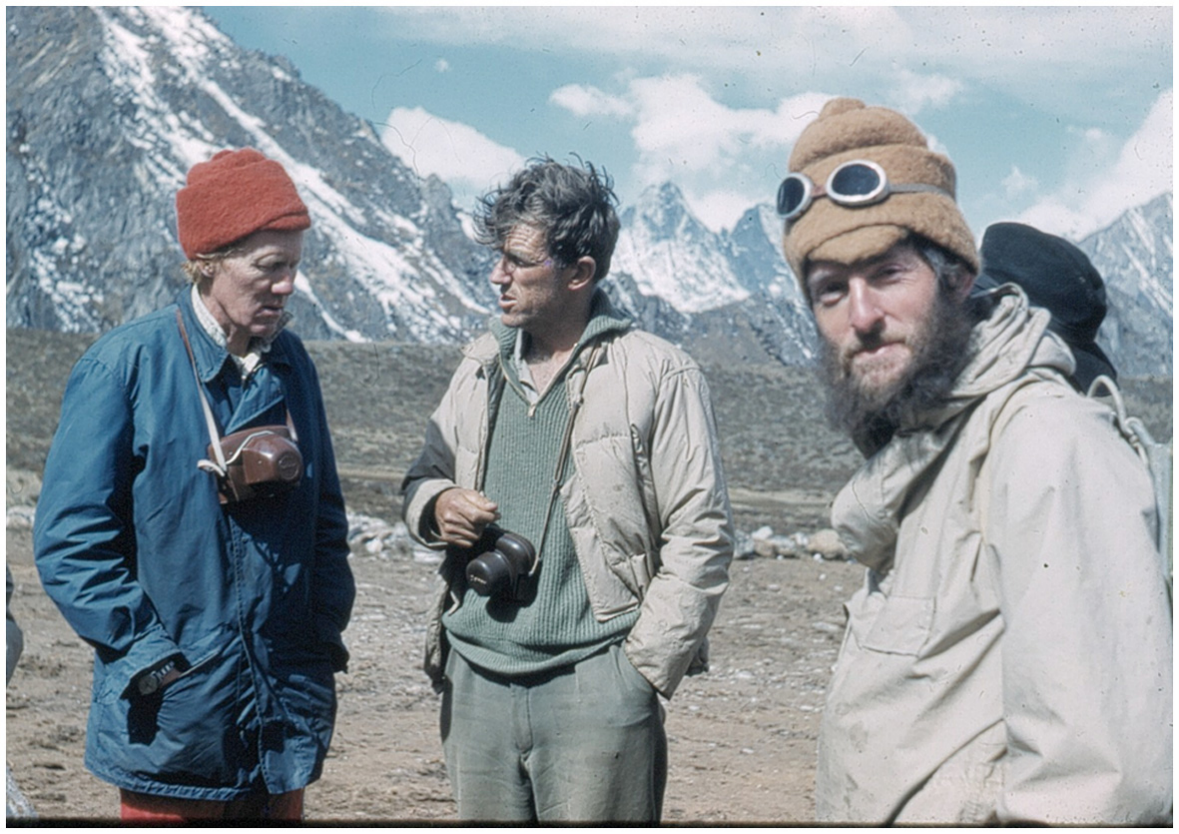
Left to right: Dr Griffith Pugh, Sir Edmund Hillary and Jim Milledge at the airstrip above our base camp (4,500 m) in Khumbu on the Silver Hut expedition 1960–1961.

In November 1959, I learned that Sir Edmund Hillary and Dr Griffith Pugh were organising an expedition to study the physiology of acclimatisation and to attempt an 8,000-m peak in Nepal. The expedition was to last 9 months, from the end of one monsoon to the beginning of the next. I wrote to Dr Pugh and, because one member dropped out, I was fortunate to get taken on. The plan was to use the autumn for setting up our base camp and high altitude stations in the Everest region (Solu Khumbu), with some members conducting a search for the Yeti. We then planned to spend the winter conducting studies on ourselves as we acclimatised; in the spring, to go over three 6,000-m passes to Makalu (8,462 m) to continue some research up the mountain and attempt to climb it. This became the Silver Hut expedition of 1960–1961 (Figure 1).

We set up the prefabricated hut on a glacier at 5,800 m where we spent the winter doing the planned physiological projects there and at our base camp at 4,500 m. On Makalu, we continued some physiology at 6,300 m and even at 7,400 m on Makalu Col, but we failed to climb the summit due to bad weather and illness. My wife Betty, an anaesthetist, worked in the Mission Hospital in Kathmandu for the 9 months we were in Solu Khumbu.

After this experience, I decided to try for a career combining research with clinical medicine, so I looked for a post in a teaching hospital. Betty and I had been keen on finding work in the Third World after our experience in Hong Kong working as volunteers in a backstreet clinic and Betty's experience in Kathmandu. When we met Dr Jacob Chandy, then principal of Vellore Christian Medical College in South India, he invited us to join the staff of this teaching hospital; we felt this was the right choice. We spent 10 years in this unique institution where I worked closely with the cardiothoracic surgeons starting open-heart surgery and the attendant intensive care, as well as with conventional respiratory medicine.

During this period, I made a number of trips to Solu Khumbu. In 1964, Dr Sukumar Lahiri, an Indian physiologist who had been on the Silver Hut expedition, organised a small physiological wing as part of Hillary's Second Schoolhouse Expedition. Our aim was to study some of the physiological differences between Sherpas and ourselves. This we did in a camp above Lukla where the expedition built an airstrip. Here, we repeated the studies I had been responsible for in the Silver Hut, on the changes in the chemical control of breathing with acclimatisation. On this occasion, we compared lowlanders (ourselves) with Sherpas and found, to our surprise, that they had significantly lower hypoxic ventilatory responses. In the middle of this period in India, we had a year's furlough, which I spent in San Francisco as a research fellow with Dr John Severinghaus at the Cardiovascular Research Institute.

We came back to the UK in 1972. I was fortunate to get a job with UK's Medical Research Council (MRC) at their newly opened Clinical Research Centre at Northwick Park Hospital, Harrow in John Nunn's Division of Anaesthesia. A year later, I took half a step sideways with a fifty-fifty contract as a consultant physician in the hospital and scientific member in the MRC. I was officially expected to spend half my time looking after patients (mainly respiratory) and half doing research. To my way of thinking, this was an ideal contract. I was fortunate in being able to keep it, despite the MRC closing the Clinical Research Centre 5 years before I retired in 1995.

During my 23 years at Northwick Park, I was able to pursue my interest in the effects of altitude on various aspects of human physiology. During the 1970s, and having failed to attract funding for any trips to the great ranges, I carried out, with a few colleagues, a series of successful field studies in the UK and Switzerland on the effects of prolonged exercise of hillwalking and altitude on fluid and electrolyte balance and the hormones controlling these. In 1981, I was fortunate to be able to make two trips on altitude, combining climbing with research. The first was to Mount Kongur (7,719 m) in Xinjiang, which was led by Michael Ward. The second was to Everest with the American Research Expedition to Everest (AMREE) led by John West. Both had been on the Silver Hut expedition. These expeditions were successful in both scientific and mountaineering terms. I was a member of two scientific expeditions with the Royal Naval and Royal Marines Mountaineering Club first to Mt. Kenya in 1987 and then to Bolivia in 1989. On these expeditions, we were able to carry out further studies on the hormones involved with fluid balance at altitude and in relation to acute mountain sickness.

**Figure 2:**
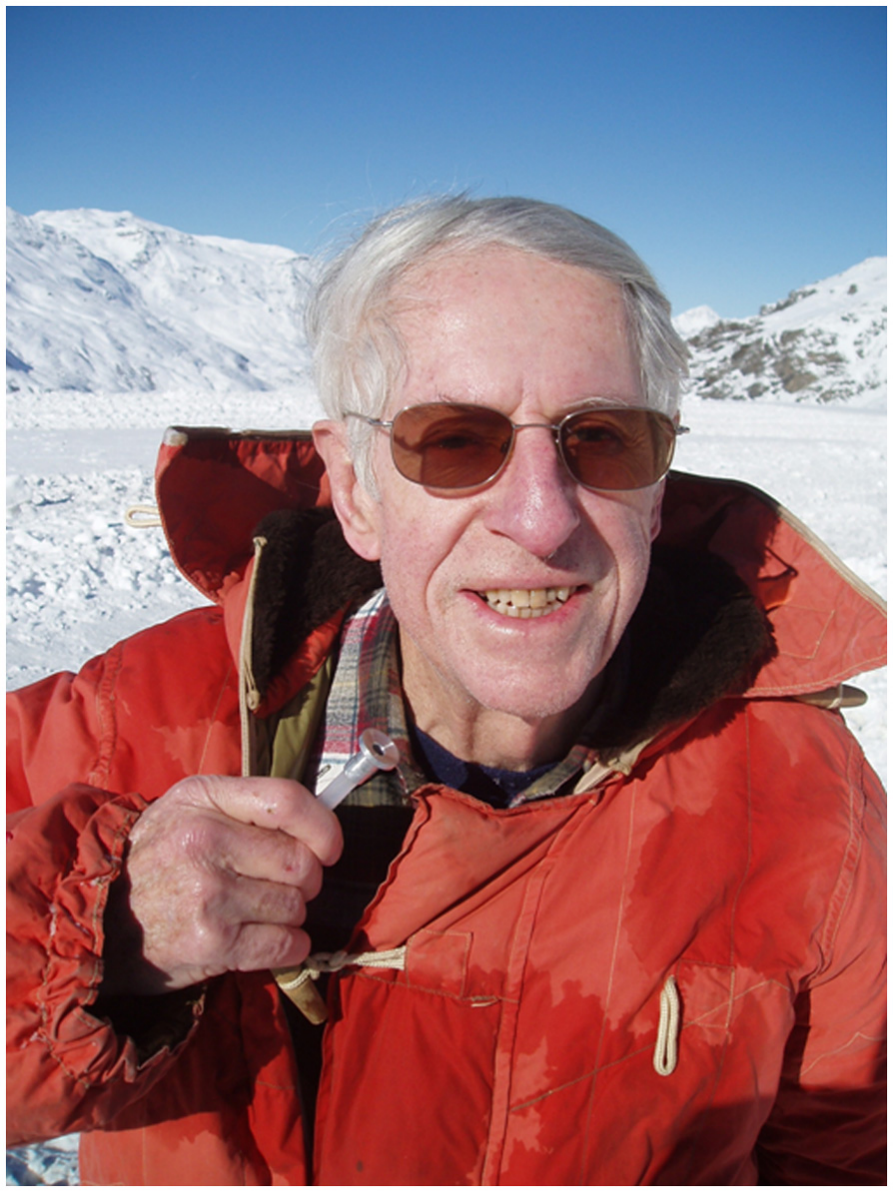
Dr Jim Milledge in 2004.

In 1992, I became the first medical director of Northwick Park Hospital. I retired from the National Health Service and MRC in 1995. Since then, I have been on two ‘Medical Expeditions’^a^—expeditions to Kanchenjunga in 1998 and to Chamlang in 2003. My last expedition was in 2010 with a team from University College London (UCL), CASE^b^ when we spent eight nights at the Margherita Hut on Monte Rosa (4,559 m). My small project was to study the effect of 30-min normoxia on hypoxic pulmonary hypertension (Figure 2).

I have lectured on various aspects of high altitude medicine and physiology to numerous groups, from undergraduates to scientific meetings as well as lay groups. In 2005, I was made honorary professor at UCL and lectured regularly for the BSc Physiology course there.

In 1989, Michael Ward, John West and I authored *High Altitude Medicine and Physiology*. The fifth edition is about to be published, and it has become the standard text in the field.

## Questions I tried to answer

Here is a list of questions I have tried to answer:

1. First set of questions:

(a) How does altitude acclimatisation change the chemical control of breathing in humans?

(b) Do highlanders (Sherpas) have a different chemical control of breathing from lowlanders?

Answers:

(a) The CO_2_ ventilatory response changes with acclimatisation so that ventilation is stimulated at a lower *P*co_2_, and the gain in the response is increased. Also, the hypoxic ventilatory response is increased.

(b) Sherpas (and Andean highlanders) have a similar CO_2_ ventilatory response to lowlanders but have a reduced hypoxic ventilatory response.

2. What is the effect of prolonged exercise (hillwalking, mountaineering) and altitude on fluid and electrolyte balance and on the hormones that control them?

Answer: Hillwalking for five consecutive days results in significant sodium retention with shifts of fluid from the intra- to extracellular compartments, including an increase in plasma volume resulting in dilutional anaemia. These changes are driven by increases in renin and aldosterone levels. Atrial natriuretic hormone levels are also increased, but any natriuretic effect is overwhelmed by the rise in aldosterone. Altitude has the effect of blunting the aldosterone response to a rise in renin. I was the unofficial leader in these studies.

3. What is the effect of altitude on electrocardiography (ECG) and of brief normoxia on hypoxic pulmonary hypertension?

Answer: The ECG shows a right axis shift with acclimatisation to altitude, and the shift increases with increasing altitude due, presumably, to the rise in pulmonary artery pressure. I was responsible for this project on the Silver Hut expedition. After only 8 h of hypoxia, the pulmonary hypertension is only *partially* relieved by 30-min normoxia. This effect of oxygen declines with increasing time spent at altitude. I led this project on the Monte Rosa expedition.

### Key people who have influenced me

Here is a list of the people who have influenced me:

1. JS Haldane has influenced me historically.

2. Three school masters—LV Turner, E Bradfield and Dr Britten.

3. A professor of medicine at the Medical School, Melville Arnott.

4. At Southampton Chest Hospital, Dr William McLeod.

5. Dr LGCE Pugh, a physiologist and scientific leader of the 1960–1961 Silver Hut expedition (Figure 1).

6. Dr John West, a physiologist and affiliated with the University of California, San Diego, a member of Silver Hut and leader of the AMREE.

7. Dr John Severinghaus an anaesthesiologist at the CVRI, San Francisco.

8. Dr John Nunn, head of the Division of Anaesthesia, CRC Northwick Park Hospital, Harrow.

9. Numerous more recent colleagues.

## My seminal paper

My seminal paper was possibly ‘Respiratory control in lowlanders and Sherpa highlanders at altitude’ which I co-authored with Sukumar Lahiri in 1967. It was published in the *Respiratory Physiology* journal.

## What is the big unanswered question in altitude acclimatisation?

Since Haldane's Pikes Peak expedition (1912), we have sought to explain acclimatisation in terms of mechanisms that increase oxygen delivery to the tissues. These may have relevance up to altitudes of about 4,000 m but seem inadequate to explain the reduced performance above this height. Manoeuvres that increase oxygen delivery do not increase performance, indicating that other factor(s) is/are inhibiting performance above 4,000 m. What are they? Possibilities include hypoxic central inhibition, increased oxygen use by the respiratory muscles or some other mechanism.

## If I were to do it all over again, I would…

The world that I grew up and in which I had my career was very different from the present. I would not change the various decisions I made during my career at all.

I consider that I have been so very fortunate in the many opportunities that have come my way and for which I have not had to strive competitively to grasp. At school, I was very happy, but being dyslexic (though the term had not been invented), I was considered just dim. However, I just scraped the necessary results to get to medical school. There, I was very average academically, until my final year when I was awarded the prize in Clinical Medicine, the only prize I ever won! Thereafter, the only credit that I can claim was that I have tried to follow my mother's aphorism, to ‘seize the opportunity of a lifetime, in the lifetime of opportunity’.

## Endnotes

^a^Medical Expeditions is a charity with the aims of furthering education and research into high altitude medicine and physiology. These aims are achieved by organising scientific and climbing expeditions at roughly intervals of 5 years and courses in mountain medicine. It also sponsors a diploma course in mountain medicine, which is now in its 12th year. I was chairman of this organisation (1996–2003) and currently a director.

^b^CASE stands for Centre for Altitude, Space and Extreme Environment Medicine. This is part of UCL with which I have been associated since 2002. Apart from under- and postgraduate teaching, it provided the leadership for a very large medical research expedition to Everest in 2007, the ‘Caudwell Xtreme Everest Expedition’ (with leader Mike Grocott). I was fortunate to be able to trek out with the main group to the base camp. The same team organised the 2010 Xtreme Alps Expedition (with leader Dan Martin) to Monte Rosa.

## Competing interests

The author declares that he has no competing interests.

